# Stereotactic radiosurgery for post operative brain metastasic surgical cavities: a single institution experience

**DOI:** 10.1186/s13014-022-02118-y

**Published:** 2022-09-26

**Authors:** Marie Cantaloube, Mohamed Boucekine, Anne Balossier, Xavier Muracciole, Mickael Meyer, Christine Delsanti, Romain Carron, Yassine Mohamed Beltaifa, Domnique Figarella-Branger, Jean Regis, Laetitia Padovani

**Affiliations:** 1 Radiotherapy Department, Assistance Publique Des Hôpitaux de Marseille, Marseille, France; 2 Unity of Research EA3279, Aix-Marseille Université, Marseille, France; 3 Department of Functional and Stereotactic Neurosurgery and Radiosurgery, Timone University Hospital, Marseille, France; 4 Department of Neurosurgery, Hôpital de La Timone, Assistance Publique-Hôpitaux de Marseille, Marseille, France; 5 Neuropathology Department, Assistance Publique Des Hôpitaux de Marseille, Marseille, France; 6 CRCM Inserm UMR1068, CNRS UMR7258 AMU UM105, Genome Instability and Carcinogenesis, Institut Paoli-Calmettes, Marseille, France; 7 Radiotherapy Department, Assistance Publique des Hôpitaux de Marseille, marseille, France

**Keywords:** Brain metastasis, Radiosurgery, Gamma Knife, Resection, Tumor bed

## Abstract

**Background:**

The standard therapy for brain metastasis was surgery combined with whole brain radiotherapy (WBRT). The latter is however, associated with important neurocognitive toxicity. To reduce this toxicity, postoperative stereotactic radiosurgery (SRS) is a promising technique. We assessed the efficacy and the tolerance to postoperative Gamma Knife radiosurgery (GK) on the tumor bed after resection of brain metastases.

**Methods:**

Between February 2011 and December 2016, following macroscopic complete surgical resection, 64 patients and 65 surgical cavities were treated by GK in our institution. The indication for adjuvant radiosurgery was a multidisciplinary decision. The main assessment criteria considered in this study were local control, intracranial metastasis-free survival (ICMFS), overall survival and toxicity.

**Results:**

Median follow-up: 11.1 months. Median time between surgery and radiosurgery: 35 days. Median dose was 20 Gy prescribed to the 50% isodose line, for a median treated volume of 5.6 cc. Four patients (7%) suffered from local recurrence. Local recurrence-free, intracranial recurrence-free and overall survival at 1 year were 97.5%, 57.6% and 62.4% respectively. In total, 23 patients (41%) suffered from intracranial recurrence outside the tumor bed. In univariate analysis: concomitant GK treatment of multiple lesions and the tumor bed was associated with a decrease in ICMFS (HR = 1.16 [1.005–1.34] p = 0.04). In multivariate analysis: a non-lung primary tumor was significantly associated with a decrease in ICMFS (HR = 8.04 [1.82–35.4] p = 0.006). An increase in performance status (PS) and in the initial number of cerebral metastases significantly reduced overall survival (HR = 5.4 [1.11–26.3] p = 0.037, HR = 2.7 [1.004–7.36] p = 0.049, respectively) and One radiation necrosis histologically proven.

**Conclusion:**

Our study confirmed that postoperative GK after resection of cerebral metastases is an efficient and well-tolerated technique, to treat volumes of all sizes (0.8 to 40 cc). Iterative SRS or salvage WBRT can be performed in cases of intracranial relapse, postponing WBRT with its potential side effects.

## Background

Currently, oncologic treatment for brain metastasis includes surgery, stereotactic radiosurgery (radiosurgery and hypofractionated external-beam radiotherapy), whole brain radiotherapy (WBRT) and systemic targeted therapy, either used alone or combined.

Care and the choice of therapy for patients suffering from brain metastasis mainly rely on the prognostic assessment of each patient. Thus, different prognostic scoring systems have been designed: RPA (Recursive Partitioning Analysis) [1], SIR (Score Index for Surgery), BSBM (Basic Score for Brain Metastases), GPA and DS-GPA (Diagnostic Specific Graded Prognostic Assessment), for which the key parameters are performance status (PS), extracranial metastases, age, primary tumor control and the number of intracranial metastases.

In the 1990’s Surgery was the recommended strategy for single, accessible lesions in the presence of neurological symptoms [2]. WBRT following surgical resection of brain metastases remained the standard treatment and current practice for many years. Nevertheless, the rate of local recurrence after surgery remains high [3].

Both the rate of recurrence and the induced large decline in neurocognition and quality of life after surgery and WBRT [4] lead to practice modifications [5]. Currently, the WBRT following surgery is challenged by SRT, stereotactic radiosurgery (SRS) or fractionated SRT (FSRT) in order to decrease the risk of neurocognitive effects with respect to overall survival for resected metastasis as reported in retrospective studies [6–8], phase 2 [9] and phase 3 trials [10].

Here we report our experience concerning adjuvant therapy of brain metastases.

The aim of our study was to retrospectively assess the efficacy of GK RS after macroscopic complete surgical resection of brain metastases in terms of local and intracranial control, and tolerance.

## Methods

### Population

Between February 2011 and December 2016, 64 patients and 65 surgical cavities were treated by GK after surgical resection. Patients were included retrospectively, from the radiosurgery center in our institution.

Indications for surgery depended on the size of the lesion, its location, the presence of neurological symptoms with a visible mass on imaging, or the need for histological evidence, as well as the disease being oligometastatic. Diagnosis of brain metastasis could be the first sign of disease.

Surgery was en bloc when possible. Only patients with complete removal of the lesion proved on the histopathology report or on postoperative imaging (brain scan or MRI) were included in the study. All types of histology and location of primary tumors were eligible. Molecular biology analysis was carried out on the primary or on resected brain metastases.

Patients may have had previous brain treatments, either radiosurgery, stereotactic radiotherapy or WBRT.

Systemic therapy for the primary tumor did not exclude them from the study.

When intracranial lesions were present at the time of GK of the tumor bed, they were treated at the same time by GK.

The decision to perform GK was taken at a multidisciplinary meeting (MM), in presence of a neurosurgeon, a radiotherapist and an oncologist.

All of the patients signed an informed consent form.

### Treatment planning and delivery

RS was performed as soon as possible after surgery, depending on the patient’s general state of health and the absence of postoperative complications.

All of the patients had a prior consultation with a radiation oncologist.

In the absence of any contraindication, all radiosurgery was performed using a model C or Perfexion GK (Elekta AB) with a stereotactic frame. Prior to treatment planning, he day of treatment, a stereotactic cranial MRI was performed with gadolinium contrast dye as well as a stereotactic injected cranial CT-scan. Treatment was planned using GammaPlan software (Elekta AB). The most frequently prescribed dose was to the 50% isodose line. The dose of radiation was calculated according to the global volume of the tumor, defined as the contrast area around the resection cavity, and to its location. No margin was added in this study***).**** Extra dura was included in the taret volume if there was pre operative contact with meningitis. No margin were added in this case.* Indeed, the GK plan was defined to include the resection cavity in the prescribed isodose. Structures at risk around the lesion were contoured.

In cases where there were several tumors (35 cases, 55%), matrices with multiple doses were used. Radiation dose planning was calculated to respect the principle of limiting the therapeutic dose to the target and ensuring the steepest gradient possible. Several isocenters were often used to achieve this. All of the patients had a stereotactic frame fitted, after local anesthesia and mild sedation. We try to perform SRS between 3 days and 3 weeks post-surgery. The median dose delivered was 20 Gy [16–26] to a median prescribed isodose line of 50% [40–55].

### Patient follow-up

After treatment, patients came to consultations at 6 weeks, 3 months and then every 3 months.

Patients were clinically examined and had a cranial MRI or CT-scan in cases where MRI was contraindicated.

### Assessment criteria

Local relapse was defined at a MM, from a cranial MRI showing an increase in the volume of the treated lesion measured at the T1 gadolinium sequence and on two consecutive exams. To differentiate between a diagnosis of radiation necrosis or pseudoprogression, if necessary, a battery of tests was performed such as multimodal MRI, 18F-FDG or -FDOPA PET scans in persisting cases of doubt or even surgical removal.

Local recurrence-free survival (LRFS) was defined as the interval between the date of RS and the date of local recurrence (within the irradiated zone). Local recurrence-free patients were censured the last time they were seen or when they died.

Intracranial metastasis-free survival (ICMFS) was defined as the interval between the date of RS and the date of intracranial relapse outside the initial target area. Intracranial relapse-free patients were counted the last time they were seen or when they died.

Overall survival (OS) was defined as the interval between the date of RS and the date of death, all causes combined. Living patients were counted the time they were last seen.

Disease-free survival (DFS) was progression-free survival, defined as the interval between the date of RS and the date of the first event (local recurrence, appearance of an intracranial metastasis outside the irradiated volume, of an extracranial metastasis or of the recurrence of the primary tumor). Disease-free living patients were counted the last time they were seen.

Clinico-radiological tolerance was assessed by the physician at each follow-up and from imaging results.

### Statistical analysis

Quantitative variables were given as the number of observations (N), median, minimum and maximum. ANOVA or the Kruskal–Wallis test (depending on the statistical hypothesis applied) was used to compare the distribution of quantitative variables when more than 2 groups were compared, and Student’s t-test or Mann–Whitney’s test was used if only 2 groups were compared.

Categorical variables were given as the number of observations (N) and the frequency (%) of each term. Missing categories were counted. Chi-squared or Fisher’s exact test was used to compare proportions (depending on the statistical hypothesis applied).

Median follow-up time was calculated using the reverse Kaplan–Meier method.

Follow-up was defined as the time between the date of RS and the date last seen, death was censured.

Kaplan–Meier’s survival curve was used to analyze survival data and to estimate rates and median times of survival. In latter cases, the survival curves used are shown.

Cox proportional hazards ratio model was used to compare survival distribution after adjustment for potential prognostic factors. Hazard Ratios (HR) and 95% confidence intervals (CI) were calculated. Variables with p < 0.2 in univariate analysis were integrated into a multivariate analysis. Given the number of patients, automatic selection methods (increase and decrease) were applied to maintain a reasonable number of variables compared to the number of patients.

Each variable added to the model was tested using Wald’s test. All tests had a 5% significance threshold. Analyses were carried out using IBM SPSS v20 software.

## Results

### Patient characteristics

In our study, 64 patients and 65 surgical cavities were analyzed. Patient characteristics are shown in Table 1.

**Table 1:** Patient characteristics*other = jejunum (1),esophagus (1), endometrium (1), ovary (1), Sarcoma (1), pancreas (1), prostate (1), thyroid (1) NSCLC = non-small cell lung carcinoma, SCLC = small cell lung carcinoma, PS = performance status

	N	%	Median	Min–Max
Gender				
Woman	33	51.6		
Man	31	48.4		
Age (years)			62.5	[21—82]
< 65 years	39	60.9		
≥ 65 years	25	39.1		
Initial PS				
0	31	56.4		
1	21	38.2		
≥ 2	3	5.4		
*Missing*	9			
Primary				
NSCLC	40	62.5		
SCLC	2	3.1		
BREAST	3	4.7		
KIDNEY	6	9.4		
Unknown	2	3.1		
Colorectal	3	4.7		
Other	8	12.9		
Extracranial metastasis				
Yes	25	44.6		
No	31	55.4		
*Missing*	8			
First brain metastasis				
Yes	24	38.1		
No	39	61.9		
*Missing*	1			
RPA class			2 [1–3]	
1	9	16.4		
2	43	78.2		
3	3	5.4		
*Missing*	9			
GPA score			2.5 [0–4]	
Lung adenocarcinoma	37	100		
Mutation KRAS	13	40.6		
Mutations EGFR	3	9.4		
Mutation HER2	1	3.1		
Translocation ALK	1	3.1		
No mutations	14	43.8		
Missing	5			
Mutation analysis				
On primary	Aug-37	21.6		
On metastasis brain	29/37	78.4		

Median follow-up of patients was 11.1 months [range 0–79.15]. Eight patients were lost to follow-up after RS. Included patients had a median age of 62.5 years at the time of brain metastasis therapy and 51% were women. They were in a good general state of health with an ECOG PS mainly below 1 (56% of cases).

The most frequently observed primary was non-small cell lung carcinoma for 40 patients (62.5%), with adenocarcinoma histopathology (37 patients, 57.8%), and 13 patients carried a KRAS mutation, 3 patients an EGFR mutation and 1 patient carried an ALK translocation, found either in the biopsy of the primary or the histology analysis of the brain metastasis (Table 1). Molecular biology analyses were mainly carried out on brain metastases (78.4%).

Brain metastasis was the first sign of cancer for 38% of patients, and 44.6% of patients had extracranial metastases at the time of treatment for brain metastasis.

A few patients had previously received brain treatment: 3 patients had received WBRT and 4 patients had undergone GK for another lesion.

### Treatment

All patients had a macroscopically total surgery. Only 11.3% of patients had “en bloc” resection.

The median diameter of lesion on preoperative imaging was 24 mm [7–70] and almost 61% were supratentorial. Median time between surgery and GK treatment was 35 days [6–181] (Table 2).

**Table 2:** Treatment and cranial lesion characteristics: 65 lesions

	N	%	Median	Min – Max
Previous WBRT				
Yes	3	4.7		
No	61	95.3		
Previous radiosurgery				
Yes	4	6.2		
No	60	93.8		
Lesion size (preoperative)			24	[7–70]
< 30 mm	32	76.2		
≥ 30 mm	11	23.8		
Missing	22			
Location				
Supratentorial	40	60.9		
Subtentorial	25	39.1		
Surgical technique				
En bloc	6	11.3		
Piecemeal	47	88.7		
*Missing*	12			
Resection quality				
Complete	65	100		
Postoperative PS				
0	14	26.4		
1	37	69.8		
Time between Surgery-GK			35	[6–181]
(days)				
Treated volume lesion 1 (cc)			5.6	[0.8–40]
Treated volume lesion 2 (cc)			11.4	11.4
Dose (Gy)			20	[16–26]
16	4	6.4		
18	18	28.6		
20	23	36.5		
22	7	11		
24	2	3.2		
26	1	1.6		
Autres (19–21-23)	8	12.7		
Prescribed isodose (%)			50	[40–55]
Number of existing lesions treated (at time of GK treatment)			1	[0–11]
Contention frame	64	100		

Only one patient had two tumor beds treated simultaneously. The median number of existing lesions treated at the same time as the tumor bed was 1 [0–11].

Median tumor bed volume was 5.6 cc [0.8–40].

### Local control

In total, among the 56 patients followed, 4 patients had local recurrence in the irradiated zone. Relapse was diagnosed from brain MRI images and 3 out of 4 patients with relapse were efficiently retreated locally, either by GK, Cyber Knife RS or surgery. Three patients underwent a relapse in the 20 Gy isodose. The 4th patient (patient # 2) developped a recurrence in border of the 20 Gy isodose, the majority of the tumor volume was included in the 10 Gy isodose.

**Fig. 1:**
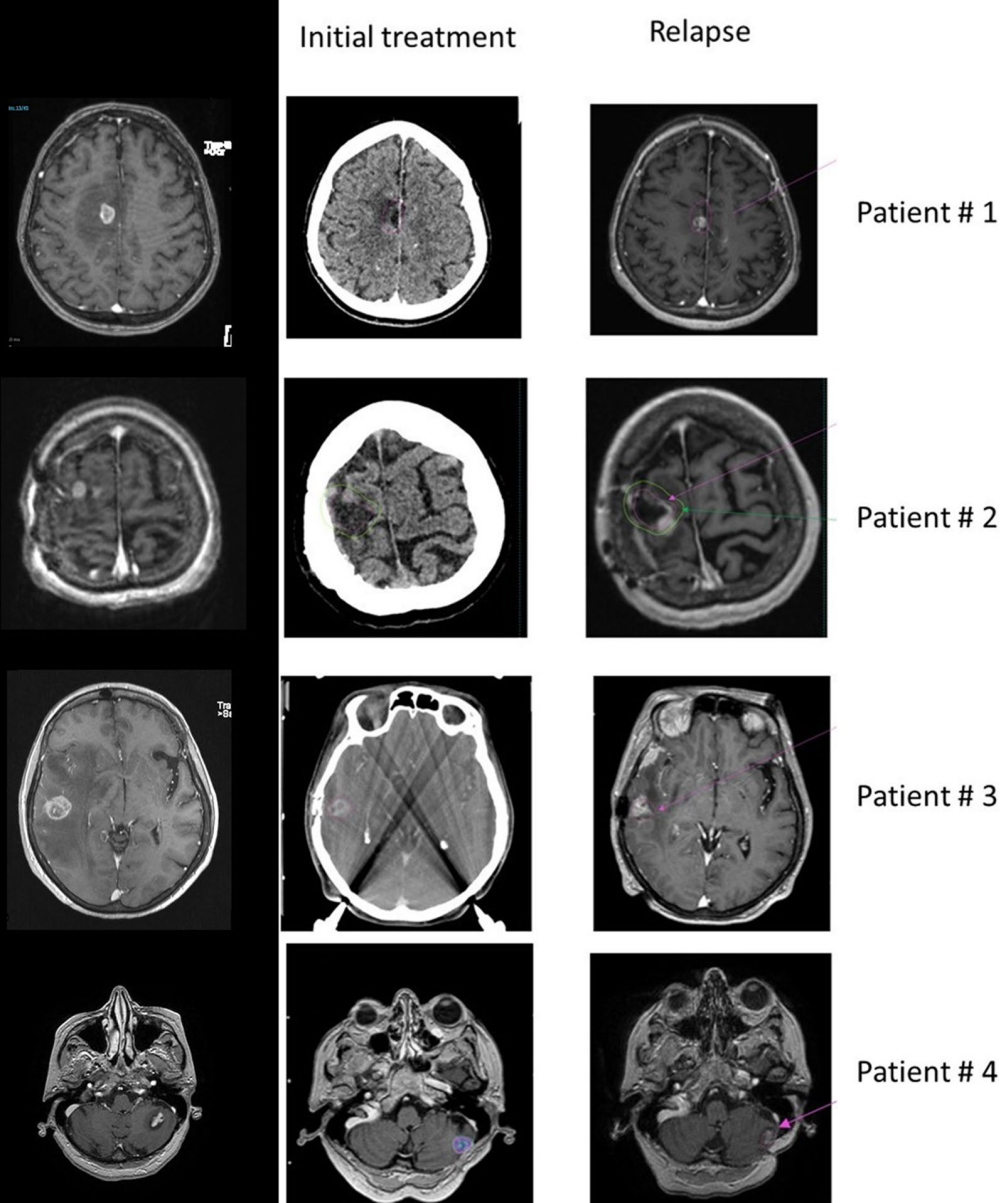
Site of recurrence according the isodoses of treatment. In pink: 20 Gy isodose. In green: 10 Gy isodose

Characteristics of these tumors are summarized in Table 3 and Fig. 1.

**Table 3:** Characteristics of the 4 tumors with local recurrence

	Dural contact	Prescribed dose (Gy/%isodose)	Volume of treatment (cc)	Primary tumor type	Delay surgery-RS (days)	Other relevant aspects
Patient #1	No	26 Gy/50%	3.5	Kidney clear cell carcinoma	28	
Patient #2	No	20 Gy/50%	3.52	Colorectal adenocarcinoma	46	RS tumor bed + 1 lesion
Patient #3	No	20 Gy/50%	4.5	NSCLC	76	
Patient #4	No	22 Gy/50%	0.8	NSCLC	139	RS tumor bed + 2 lesions 2-piece surgery

The population’s local control rate was 93%.

Median time to local recurrence was 25.9 months [5.6–36.3].

**Fig. 2:**
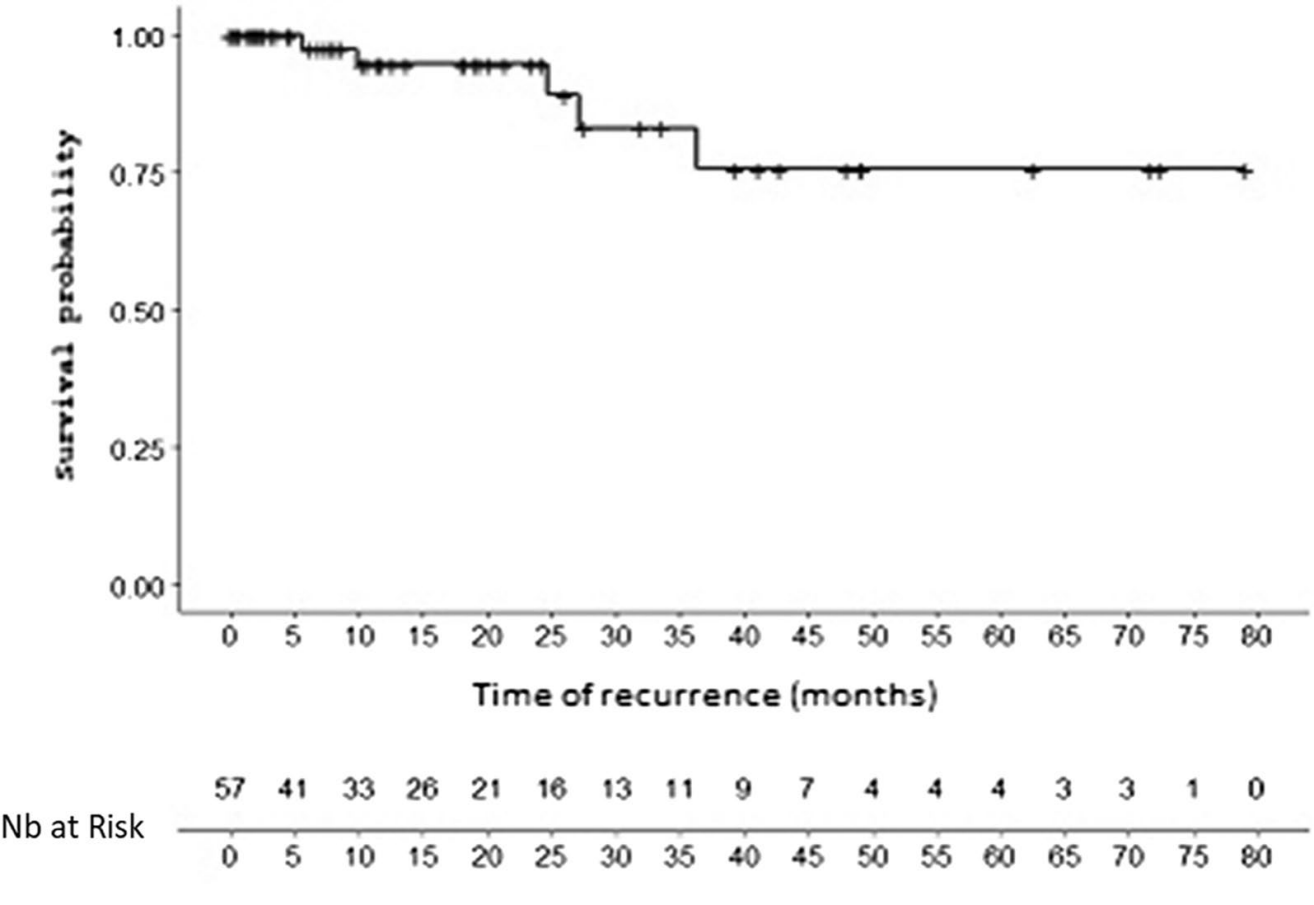
Local recurrence-free survival

Local recurrence-free survival at 6 months, 12 months and 24 months was stable at 97.5% [83.2–99.7]. The median was never reached. The local recurrence-free survival curve is shown in Fig. 2.

### Toxicity

No grade 5 toxicity was observed

One patient suffered from radiation necrosis, revealed by a period of confusion combined with hemiparesis. Histology of the surgical specimen confirmed the diagnosis of radiation necrosis. The initial lesion diameter was 30 mm across, a 20 Gy dose to the 45% isodose line had been delivered to the tumor bed, cavity volume was 14.5 cc. Radiation necrosis was diagnosed 11 months after radiosurgery therapy.

One patient had panhypopituitarism from the treatment of a lesion located in the sella turcica. Another suffered from epilepsy upon GK local recurrence salvage treatment. Finally one patient suffered from scotoma (initial lesion located in the right occipital lobe).

Otherwise tolerance was correct, no patients suffered from cerebral edema equal to or above grade 2, or from cerebral hemorrhage.

### Outcome

**Fig. 3:**
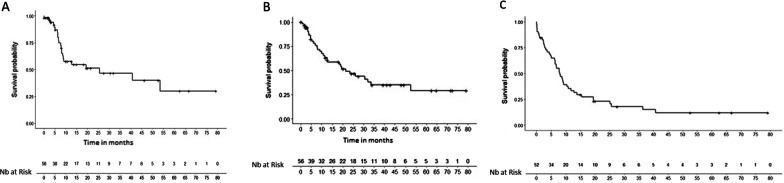
Distant intracranial progression-free survival **A**, overall survival **B**, disease-free survival **C**

Survival data are given in Fig. 3.

The rate of intracranial metastasis-free survival (ICMFS) outside the irradiated volume was 87% [73.1–94] at 6 months and 57.6% [41.2–71] at 12 months. Median ICMFS was 25.6 months.

In total 23 patients (41%) relapsed, 20 with parenchymal lesions and 3 with carcinomatous meningitis. Among these patients, 5 had two relapses and one patient had 4 relapses. Median time to the first intracranial relapse was 7.2 months.

Salvage treatment for most patients was GK on new lesions (12 patients, one of whom also had surgery), 2 patients had Cyber Knife RS and 1 patient only had surgery.

Only 3 patients had WBRT (one of whom also had surgery).

The overall survival rate was 79.6% [65.3–88.5] at 6 months and 62.4% [47.2–74.4] at 12 months. Median overall survival was 21.4 months [14.1–28.6]. In total, 30 patients died (53.5%). The rate of disease-free survival was 64.9% [50.1–76.3] at 6 months and 33.5% [21.1–46.4] at 12 months. Median disease-free survival was 7.8 months [5.4–10.3].

### Statistical analysis

Simultaneous treatment of existing lesions as well as the tumor bed with GK was associated with a decrease in distant intracranial relapse-free survival in univariate analysis (HR = 1.16 [1.005–1.34] p = 0.04). The GPA score was close to being significant (HR = 0.59 p = 0.06). In multivariate analysis, the location of the primary in “other” compared to the lung was associated with a higher risk of intracranial recurrence (“other” = GI tract, gynecological, thyroid, prostate, sarcoma (HR = 8.04 [1.82–35.4] p = 0.006)) (Table 4).

**Table 4:** Univariate and multivariate analyses concerning ICMFS: PS = performance status, GK = gamma knife, WBRT = whole brain radiotherapy. Other = Colon (1), jejunum (1), rectosigmoid (2), esophagus (1), endometrium (1), ovary (1), Sarcoma (1), pancreas (1), prostate (1), thyroid

	HR	CI 95%	P value
*Univariate analysis*			
Initial PS	0.86	0.37–1.97	0.72
Primary			
Kidney vs lung			
Other vs lung			
Extracranial metastasis at diagnosis			0.54
No	1		
Yes	1.31	0.54–3.19	
Brain metastasis as first sign of cancer			
No			0.37
Yes	1		
	1.44	0.63–3.29	
Number of initial brain metastases	1.44	0.93–2.23	0.09
Previous GK treatment	1.13	0.26–4.94	0.86
Previous WBRT treatment	0.53	0.07–4.05	0.54
BSBM	0.78	0.50–1.22	0.28
GPA	0.59	0.34–1.03	0.06
RPA	0.99	0.37–2.62	0.98
Age at time of treatment	0.99	0.94–1.03	0.73
Subtentorial location	1		0.23
Supratentorial location	1.76	0.69–4.48	
Size of preoperative lesion	1.63	0.51–5.14	0.4
Time between surgery-GK	0.99	0.97–1.00	0.32
Volume lesion treated with GK	0.91	0.83–1.01	0.09
Dose GK treatment	1.12	0.94–1.33	0.19
Other existing lesions treated at the same time with GK			
	1.16	1.005–1.34	0.04
*Multivariate analysis taking variables with p < 0.2 in univariate analysis + use of Increasing automatic selection technique*			
Primary lesions			
Kidney vs lung	1.04	0.23–4.69	0.95
Other vs lung	8.04	1.82–35.4	0.006
Other existing lesions treated at the same time with GK	1.17	1.00–1.37	0.05

With regards to overall survival, in univariate analysis an increase in BSBM and GPA scores was associated with better overall survival (HR = 0.48 [0.31–0.73] p = 0.001 and HR = 0.47 [0.28–0.78] p = 0.004, respectively). An increase in the diameter of the preoperative lesion, the presence of extracranial metastases at the time of diagnosis of brain metastasis, and a primary located in the category “other” (one patient for each of the following locations: endometrium, ovary, prostate, thyroid, sarcoma, jejunum, esophagus and pancreas) also appear to be prognostic factors (HR = 2.55 [1.02–6.4] p = 0.046; HR = 2.3 [1.06–4.96] p = 0.03; HR = 3.68 [1.01–13.35] p = 0.047, respectively), associated with a decrease in overall survival.

In multivariate analysis, an increase in initial PS and the number of initial brain metastases were associated with a decrease in overall survival (HR = 5.4 [1.11–26.3] p = 0.037 and HR = 2.7 [1.004–7.36] p = 0.049, respectively). Carrying a KRAS mutation (compared to no mutation) appeared to be a prognostic factor improving overall survival (HR = 0.066 [0.007–0.66] p = 0.021). (Table 5).

**Table 5:** Univariate and multivariate analyses concerning overall survival

	HR	CI 95%	P value
*Univariate analysis*			
Initial PS	1.53	0.93–2.50	0.08
*Primary*			
Kidney vs lung	1.11	0.38–3.27	0.84
Other vs lung	3.68	1.01–13.35	0.047
Colorectal vs lung	8.28	0.80–84.95	0.075
*Mutations Mol.Biol*			
KRAS vs no mutation	0.22	0.06–0.80	0.02
EGFR vs no mutation	0.5	0.10–2.53	0.4
HER2 vs no mutation	0.27	0.03–2.32	0.23
*Extracranial metastasis at diagnosis*			0.03
No	1		
Yes	2.3	1.06–4.96	
*Brain metastasis as first sign of cxancer*			0.8
No	1		
Yes	0.91	0.44–1.88	
Number of initial brain metastases	1.41	0.94–2.14	0.096
Previous GK treatment	0.44	0.06–3.31	0.43
Previous WBRT tretament	0.76	0.17–3.21	0.7
BSBM	0.48	0.31–0.73	0.001
GPA	0.47	0.28–0.78	0.004
RPA	2.05	0.86–4.87	0.1
Age at time of treatment	0.97	0.93–1.02	0.31
Subtentorial location	1		0.39
Supratentorial location	1.44	0.61–3.37	
Size of preoperative lesion	2.55	1.02–6.40	0.046
Time between surgery-GK	0.98	0.96–1.00	0.06
Volume of lesion treated with GK	0.98	0.93–1.04	0.68
Dose GK treatment	1.03	0.88–1.21	0.67
Other existing lesions treatedat the same time withGK moment du GK	1.15	0.87–1.50	0.3
*Multivariate analysis taking variables with p < 0.2 in univariate analysis*			
Initial PS	5.4	1.11–26.31	0.037
Nb of brain metastasis at diagnosis	2.72	1.004–7.36	0.049
Kras Mutation vs no mutation	0.066	0.007–0.66	0.021
EGFR Mutation no de mutation	6.99	0.52–93.18	0.14
HER2 Mutation no de mutation	1.88	0.11–31.56	0.65
BSBM	0.53	0.79–3.59	0.51
GPA	1.52	0.16–13.78	0.7
Delay between surgery -GK	0.97	0.93–1.01	0.23
Extra brain metastasis at diagnosis	0.6	0.1–3.62	0.58

The KRAS mutation being mainly present in lung adenocarcinoma (13/16 for the lung, 2/16 for the rectosigmoid and 1/16 for the jejunum), we performed a multivariate analysis on just the lung patient population. In this group of patients, the presence of the KRAS mutation compared to no mutations was associated with better overall survival in univariate (HR = 0.21 [0.05–0.78] p = 0.02) and multivariate analyses (HR = 0.15 [0.03–0.81] p = 0.028).

Prognostic factors for local control were not analyzed, given the small number of events.

## Discussion

This single-center, retrospective study confirms the efficacy and correct tolerance of GK treatment as adjuvant therapy for brain metastasis tumor beds.

The most frequently prescribed dose was 20 Gy to the 50% isodose line, for a median treated volume of 5.6 cc.

We observed an excellent rate of local control that reached 93%. Only 4 patients suffered from local relapse, 3 of whom received local salvage treatment.

This control rate is similar to or even higher than those previously published. Indeed, many retrospective studies [6–8] and one prospective phase 2 trial [9, 10] reported 1-year local control rates of 70 to 90% with 10 to 20 months of median survival,. In the meta-analysis by *Gans *et al. [11], covering 14 studies and 629 patients, the 1-year local control rate was 85%.

The difference between our results and some other retrospective studies with lower local control rates, can be partly explained by the lack of distinction between pseudo-progression and local recurrence [12].The authors have shown that a significant proportion of patients treated with SRS have demonstrated tumor enlargement that do not necessary represent a tumor relapse.

We diagnosed local recurrence in MM with a range of arguments and access to a 18F-FDG or -FDOPA PET scan in case of doubt, which was not always the case in some studies where they only performed cranial MRI, [13, 14]. Furthermore, treatment doses were also different. Indeed, we used a median dose of 20 Gy to the 50% isodose line while in studies by *Jensen *et al.,* Iwai *et al.and *Choi *et al., for respective local control rates of 62.3%, 76% and 76%, the respective treatment doses were 17 Gy, 17 Gy and 15 Gy prescribed to the 50% isodose line [13, 15].

Different prognostic factors have been found in several studies: a cerebral lesion with a diameter of more than 3 cm predicted local relapse [14] or a larger tumor was associated with a shorter interval before local recurrence [16]. On the contrary, *Minitti *et al. [17] reported local control rates comparable to those published despite preoperative lesions measuring over 3 cm.

In our study, there was no size limit for lesions (from 7 to 70 mm) or for treated volumes (from 0.8 to 40 cc). In multivariate analysis, we found that the size of tumor or its location in the brain had no impact. Furthermore, the median time between surgery and RS in this study was 35 days which may partly explain the high level of local control reported, as it has been clearly noted that an interval exceeding 3 weeks had a negative impact on local control [18–20]

In our study, 41% of patients had intracranial relapse, with a median time of occurrence of 7.2 months, These results are comparable to published data with local relapse rates between 35 and 63% depending on the study [5, 13, 17]. In our study we report no leptomeningeal dissemination.

Most of the patients with relapse had GK as salvage treatment, only three had WBRT. This highlights the possibility of performing focal treatment several times on lesions in different places, and enables to delay WBRT even longer.

With the aim of reducing the risk of leptomeningeal spread [21] neoadjuvant SRS is currently under scrutiny. Four trials are currently underway: one phase 2 trial (NCT03368625) and three phase 3 trials in order to compare pre- versus post-operative SRS for operable metastases (NCT03750227, NCT04069910, NCT03741673), for sizes between 2 and 7 cm depending on the number of fractions planned.

Furthermore, investigations into a synergy of a combination of RS and targeted therapies could improve local and distant tumor control.

Median overall survival was 21.4 months, apparently higher than other retrospective studies. Surprisingly, the presence of a KRAS mutation was one of the factors associated with better prognosis in multivariate analysis. This mutation is usually known to be associated with poor prognosis, especially for lung adenocarcinoma [22]. We analyzed the subgroup with primary cancer located in the lung and this confirmed our results. In scientific literature, even in populations with primary cancer mainly located in the lung, molecular biology results have never been studied as risk factors in the context of SRS treatment of surgical cavities.

Nevertheless, a few studies investigated into the impact of mutational status for patients with non-small cell lung carcinoma (NSCLC) having irradiation of existing brain metastases, and they mostly reported the negative impact of KRAS mutation on local, intracranial and overall relapse [23, 24]. On the contrary, EGFR and ALK mutations were reported to be factors of improved overall survival thanks to the administration of targeted therapy [23]. In our study, patients suffering from NSCLC carrying the KRAS mutation showed a better overall survival than patients with no mutations: over 1/3 of patients carrying the KRAS mutation were given immunotherapy while it was given to less than 10% of the rest of the population (data not shown). This systemic care could partly explain these unexpected results, due to the synergy immunotherapy/high dose radiotherapy [25–27]. These results deserve to be studied in further detail in future studies.

We reported a very low rate of toxicity, with only one patient suffering from radiation necrosis. The incidence of radiation necrosis following SRS, hypofractionated or not, is very variable and under controversy, with rates varying from 0 to 20% in literature, and reported risk factors being the size of the preoperative lesion and the volume of the irradiated cavity > 3 cm [28–30].

A phase 3 randomized trial currently underway (NCT04114981), is comparing RS and hypofractionated SBRT for volumes ≥ 2 cm and < 5 cm.

In this context, different others modalities of adjuvant radiotherapy are currently assessed in a goal to improve local control and/ or reduce brain toxicity such as intraoperative radiotherapy or intra operative brachytherapy; several published studies have reported a high variability in term of one-year-local control, from 50% [31] to 88% with a low rate of radionecrosis (7%) [32]. Larger prospective studies are needed to confirm these favorable results including combination with systemic treatment [33]. The role of a novel collagen Tile cesium brachytherapy is currently in tested. It has been recently reported, about 11 ptients treated for 16 brain metastasis an 1 –year-local control at 100% and no radionecrosis [34].

Based on these results regarding the low rate of toxicity and the excellent local control, we advise to deliver 20 Gy prescribed at 50% isodose avoiding extra margin.

## Conclusion

To conclude, we have reported our experience of GK treatment as adjuvant therapy of brain metastasis tumor beds with no limit in size or treated volume. Our results reveal an excellent rate of local control and correct tolerance, with only one case of radiation necrosis.

Studies that are currently underway shall probably enable us to standardize treatment practice, according to the size, to fractionation and to the pre- or postoperative timing of stereotactic uni- or mullti-fractionated radiotherapy. Investigations into the synergy of a combination of RS and targeted therapy, could further improve local and distant tumor control. 

## Data Availability

The datasets used and/or analysed during the current study are available from the corresponding author on reasonable reques.

## Abbreviations

BSBM: Basic score for brain metastases
CK: Cyber knife
DFS: Disease-free survival
DS GPA: Diagnostic-specific graded prognostic assessment
EANO: European Association of Neuro-Oncology
GK: Gamma knife
GPA: Graded prognostic assessment
ICMFS: IntraCranial metastasis-free survival
LRFS: Local relapse-free survival
LS: Leptomeningeal spread
MM: Multidisciplinary meeting
NSCLC: Non-small cell lung carcinoma
OS: Overall survival
PS: Performance status
RS: Radiosurgery
RPA: Recursive partitioning analysis
RT: Radiotherapy
RTOG: Radiation Therapy Oncology Group
SIR: Score index for radiosurgery
VMAT: Irradiation with Volumetric Modulated Arc Therapy
WBRT: Whole brain radiotherapy
